# NRGSuite-Qt: a PyMOL plugin for high-throughput virtual screening, molecular docking, normal-mode analysis, the study of molecular interactions, and the detection of binding-site similarities

**DOI:** 10.1093/bioadv/vbaf129

**Published:** 2025-06-16

**Authors:** Gabriel Tiago Galdino, Thomas DesCôteaux, Natalia Teruel, Rafael Najmanovich

**Affiliations:** Department of Pharmacology and Physiology, Université de Montréal, Montréal, QC H3C 3J7, Canada; Department of Pharmacology and Physiology, Université de Montréal, Montréal, QC H3C 3J7, Canada; Department of Pharmacology and Physiology, Université de Montréal, Montréal, QC H3C 3J7, Canada; Department of Pharmacology and Physiology, Université de Montréal, Montréal, QC H3C 3J7, Canada

## Abstract

**Summary:**

We introduce NRGSuite-Qt, a PyMOL plugin, that provides a comprehensive toolkit for macromolecular cavity detection, virtual screening, small-molecule docking, normal mode analysis, analyses of molecular interactions, and detection of binding-site similarities. This complete redesign of the original NRGSuite (restricted to cavity detection and small-molecule docking) integrates five new functionalities: protein–protein and protein–ligand interaction analysis using Surfaces, ultra-massive virtual screening with NRGRank, binding-site similarity detection with IsoMIF, normal mode analysis using NRGTEN, and mutational studies through integration with the Modeler Suite. By merging these advanced tools into a cohesive platform, NRGSuite-Qt simplifies visualization and streamlines complex workflows within a single interface. Additionally, we benchmark a newer version of the Elastic Network Contact Model (ENCoM) for normal mode analysis method, utilizing the same 40 atom-type pairwise interaction matrix that is used in all other software. This version outperforms the default model in multiple benchmarking tests.

**Avalilability and implementation:**

The Installation guide and tutorial is available at https://nrg-qt.readthedocs.io/en/latest/index.html. The NRGSuite-Qt is implement in Python.

## 1 Introduction

Computational methods for evaluating protein structures and complexes are fundamental to drug discovery and understanding the mechanisms of various biological systems. However, studying macromolecular dynamics using traditional all-atom models can be extremely costly, depending on the size of the system studied and the amplitude of the movements of interest (Kmiecik *et al.* 2016), leading to simulation runtimes ranging from days to weeks on expensive high-performance computing clusters (estimated to yield 1–5 ns per dollar) (Abraham *et al.* 2015, Kutzner *et al.* 2022). Recent advances in protein structure prediction and the wide availability of purchasable synthetic compounds have increased the demand for fast and reliable tools capable of modeling and comparing millions (or even billions) of systems at reduced computational costs (Volochnyuk *et al.* 2019, Jumper *et al.* 2021). Methods using coarse-graining and simplified representations of atoms, residues, and interactions offer an efficient alternative to traditional methods, reducing complexity and enabling the study of large biological systems in a shorter times without significant loss in accuracy. For example, to evaluate the binding affinity prediction of a dataset of 23 SARS-CoV-2 Spike Receptor Binding Domain (RBD) complexes, Surfaces achieves similar performance to free energy perturbation (FEP) calculations derived from 100 ns molecular dynamics simulations while operating on a timescale of minutes instead of weeks per structure (Sergeeva *et al.* 2023, Teruel *et al.* 2023). Additionally, Surfaces was run on a single laptop CPU core, compared to the 4 graphical processing units (GPUs) required for FEP (estimated to cost 20–100 dollars per RBD complex).

With increased maturity, molecular docking and docking-based virtual screening has become a valuable tool in drug discovery. This approach uses scoring functions to enrich true binders in ligand libraries for targets with known or predicted structures (Agu *et al.* 2023). The Flexible Artificial Intelligence Docking (FlexAID) software (Gaudreault and Najmanovich 2015), previously introduced by our group, performs the docking of flexible ligands while also allowing flexibility in the target’s side chains. It has been extensively validated in various scenarios (Duchêne *et al.* 2014, Pandey *et al.* 2022) and was previously available in the original NRGSuite as a PyMOL plug-in for real-time docking (Gaudreault *et al.* 2015). NRGSuite-Qt reintroduces an intuitive, user-friendly, and interactive interface for performing docking simulations for single ligands employing FlexAID. It also contains GetCleft (Gaudreault and Najmanovich 2015), a software for cavity detection and binding site definition based on the SurfNet algorithm (Laskowski 1995), providing seamless integration between cavity determination and the exploration of these cavities for small-molecule docking.

One recent FlexAID application was the identification of ligands targeting the Ebola VP35 protein in a virtual drug repurposing screening campaign. In this workflow, ligands from the Chemical Component Dictionary (CCD) (Westbrook *et al.* 2015) were first screened using FlexAID, followed by selection of top ranked docked ligands using binding-site similarity analysis with IsoMIF (Chartier and Najmanovich 2015), a software for binding-site similarity calculation based on the detection of binding site molecular interaction field similarities—also integrated in NRGSuite-Qt. Two molecules were identified to be capable of preventing the interaction between VP35 and ubiquitin, reducing viral replication both *in vitro* and *in vivo* (Rodríguez-Salazar *et al.* 2024). The fact that binding sites known to bind these top ranked molecules are more similar to the target binding site than expected by chance may suggest that experimentally observed interactions important for binding these molecules are present in the target binding site and exploited by the docking software—a hypothesis that can be validated using Surfaces. Another noteworthy application was in the identification of Toyocamycin as a selective CDK9 inhibitor in cancer cells, with a binding site characterization that opens the possibility for the design of novel CDK9 inhibitors (Pandey *et al.* 2022).

The FlexAID scoring function (FSC) relies on a pairwise interaction matrix comprising 40 SYBYL-based atom types. This interaction matrix was derived through Monte Carlo optimization based on known ligand–protein crystal structures available in the Protein Data Bank (PDB) and shows a strong correlation with established chemical data (Gaudreault and Najmanovich 2015). The FSC was implemented in Surfaces (Teruel *et al.* 2023), a method for analysing protein–protein (PPI) and ligand–protein (LPI) interactions. Surfaces has demonstrated performance comparable or superior to molecular dynamics–based methods (e.g. MM/PB-SA, MM/GB-SA, FEP+, and gRINN analysis) in predicting the effects of mutations on PPI and binding affinity, particularly in evaluating the impact of mutations on interactions involving the SARS-CoV-2 Spike protein. This software was also successfully used to identify mutations that disrupted interactions between ligands and VP35 without affecting its interaction with ubiquitin (Rodríguez-Salazar *et al.* 2024).

NRGRank (Descoteaux and Najmanovich 2025), a method for ultra-massive virtual screening, uses a simplified version of FSC. It is a structurally informed ultra-massive virtual screening software that can screen large ligand libraries at extremely low computational cost resulting in a ranked list of ligands and approximate poses (actual binding poses can be obtained subsequently with FlexAID). This software was validated in different scenarios and is capable of screening 50 000 ligands per hour on a common laptop (8 cores).

More recently, we employed the FSC within Elastic Network Contact Model (ENCoM) (Frappier and Najmanovich 2014) as part of the Najmanovich Research Group Toolkit for Elastic Networks (NRGTEN) (Mailhot and Najmanovich 2021). NRGTEN was used to calculate entropic dynamic signatures (EDS), which are vectors of predicted structural fluctuations per residue, for the docking complexes of the μ-opioid receptor with ligands of known efficacy generated by FlexAID. Subsequently, these EDS were used to train a LASSO linear regression-based efficacy predictor, which demonstrated strong predictive power and identified residues important for receptor activation (Galdino *et al.* 2024).

In this work, we introduce *NRGSuite-Qt*, a PyMOL plugin, inspired by the first version of NRGSuite (Gaudreault *et al.* 2015). The NRGSuite-Qt, like its predecessor that is no longer functional, gives access to GetCleft, a method to detect and refine cavities in macromolecular structures, and to FlexAID, a molecular docking software (Gaudreault and Najmanovich 2015). Additionally, NRGSuite-Qt also gives access to NRGRank (Descoteaux and Najmanovich 2025) virtual screening simulations, the analysis of ligand–protein and protein–protein interactions with Surfaces (Teruel *et al.* 2023), NRGTEN (Mailhot and Najmanovich 2021) for the calculation of dynamical signatures, generation of conformational ensembles and the effect of mutations on dynamics and stability, as well as IsoMIF (Chartier and Najmanovich 2015) for the detection of binding-site similarities. We also perform a comprehensive evaluation of NRGTEN/ENCoM with the SYBYL-based atom types used in FlexAID and support the interpretation of IsoMIF results with distributions of Tanimoto scores of binding-site similarities.

## 2 Overview

The older version of NRGSuite was based on Tkinter, the previous platform for PyMOL plug-ins, and was limited to GetCleft and FlexAID for real-time docking. In contrast, NRGSuite-Qt not only includes GetCleft and FlexAID but also provides a user-friendly interface for additional tools developed by the Najmanovich Research Group: NRGRank, Surfaces, NRGTEN, IsoMIF, and an optional Single Mutations functionality using the well-established Modeler software (Šali and Blundell 1993). Descriptions of each software, along with their original references and applications, are provided in Table 1. NRGSuite-Qt is designed to support both individual and combined use of these tools, allowing outputs from one software to be used as inputs for another, thereby enabling users to create various distinct workflows.

**Table 1.** Overview of tools incorporated in the NRGSuite-Qt plugin.

Tool name	Functionality	Reference
① **GetCleft**	Finds cavities (clefts) in protein structures. It can define specific clefts around ligands/residues or multiple clefts in the structure. Different clefts can be defined using variations in the cleft detection parameters. Defined clefts can be refined interactively within PyMOL. It is commonly used to define binding sites for FlexAID, NRGRank, and ISOMIF.	Gaudreault and Najmanovich (2015)
② **NRGRank**	Performs ultra-massive high-throughput screening simulations. It supports screening across predefined datasets, including FDA-approved drugs (2500 ligands), the Chemical Component Dictionary (33 000 ligands), and all-tetrapeptides (16 000 ligands). It also allows the user to upload a SMILES ligand file. NRGRank can screen around 50 000 ligands per hour (8 cores).	Descoteaux and Najmanovich (2025)
③ **FlexAID**	A molecular docking tool for flexible ligand docking into protein targets, accounting for solvent interactions implicitly. It provides realistic docking poses and is commonly used in combination with NRGTEN to dock ligands on individual structures within conformational ensembles and surfaces for ligand–protein interaction analysis.	Gaudreault and Najmanovich (2015)
④ **Surfaces**	Computes pairwise molecular interaction analysis based on the calculation of surface areas in contact. This tool can be applied to *protein–ligand* and *protein–protein* interaction studies of crystal structures, computational models, and docking complexes. It is commonly used for the analysis of FlexAID results and Single Mutants.	Teruel *et al.* (2023)
⑤ **NRGTEN**	Uses Normal Mode Analysis for the study of dynamics of proteins and protein–ligand complexes. It can calculate dynamical signatures of the system, giving an overview of the flexibility in each residue. These signatures can be employed to study the effect of ligand binding and mutations on the overall flexibility of proteins and can be used in combination with FlexAID and Single Mutations tool.	Mailhot and Najmanovich (2021)
⑥ **Single Mutations**	Optional feature on NRGSuite-Qt to model single mutations using Modeller upon licensing. These mutants can be used as input for Surfaces and NRGTEN.	Eswar *et al.* (2006)
⑦ **IsoMIF**	Identifies molecular interaction field similarities between protein binding sites defined with GetCleft. It detects both geometric and chemical equivalences across cavities using six chemical probes. This tool can be used to predict protein function, detect potential off-targets, suggest bioisosteric replacements, and in drug repurposing efforts.	Chartier and Najmanovich (2015)

For virtual screening, three pre-processed ligand datasets are provided by default: the majority of CCD (Westbrook *et al.* 2015), the PDB chemical compound dictionary of all non-macromolecular (protein or nucleic acid) molecules currently in PDB structures, FDA-approved drugs available in DrugBank (Knox *et al.* 2024), and tetra-peptides made by Prasasty and Istyastono (2019). Each software is designed to use structures loaded in the PyMOL interface, and all resulting structures are automatically loaded into the PyMOL interface to facilitate software integration.

Optionally, we provide functionality for creating single mutants using Modeler (Šali and Blundell 1993, Eswar *et al.* 2006), based on their mutate_model script (Sali and Webb 2022). This functionality can be accessed through an Anaconda (Anaconda, Inc. 2025) installation of the NRGSuite-Qt, but users need to procure and install their personal Modeler license.

No additional benchmarking is necessary for most of the software implemented in the NRGSuite, as the tools have already been extensively validated in the methods’ original publications (see the references in Table 1). The only exception to this is ENCoM, since the version available in NRGSuite-Qt as NRGTEN was implemented using the 40 atom types and the respective pairwise pseudo-energy interaction matrix of FlexAID, instead of the eight atom types and respective interaction matrix used in the original ENCoM implementation (Frappier and Najmanovich 2014). This new implementation was partially validated in the work of Galdino *et al.* (2024) for the prediction of ligand efficacy in the μ-opioid receptor. However, that validation used a different subset of benchmarks from those used for the original ENCoM implementation and from the work of Mailhot *et al.* (2022) for simulating RNA dynamics and could lead to significant differences in performance compared to the original, validated version. Therefore, we conducted a comprehensive analysis of the performance of this new version of ENCoM across all previously developed benchmarks reported in our Supplementary Materials, available as supplementary data at *Bioinformatics Advances* online. We did not observe any loss in performance for the new version and in fact observe mild gains for certain benchmarks.

Additionally, we calculated the distribution of Tanimoto coefficients of binding-site similarity for all combinations of the 102 targets from the DUD-E dataset (Mysinger *et al.* 2012) to aid in the interpretation of IsoMIF results. The analysis includes comparisons within and between protein families, such as kinases, proteases, nuclear receptors, and others, as classified by Mysinger *et al.* (2012). As expected, we observe a shift in the mean Tanimoto coefficient for proteins within the same family, particularly kinases, which exhibit higher similarity within the ATP binding site. These distributions serve as a reference for computing the *Z*-score and *P*-value of IsoMIF results, allowing users of NRGSuite-Qt to assess the statistical significance of detected binding-site similarities, similarly to its implementation in the Ebola VP35 study (Rodríguez-Salazar *et al.* 2024). A detailed discussion of the distributions, including family-specific comparisons and statistical trends, is provided in the Supplementary Material, available as supplementary data at *Bioinformatics Advances* online.

## 3 Case studies

**Figure 1.**
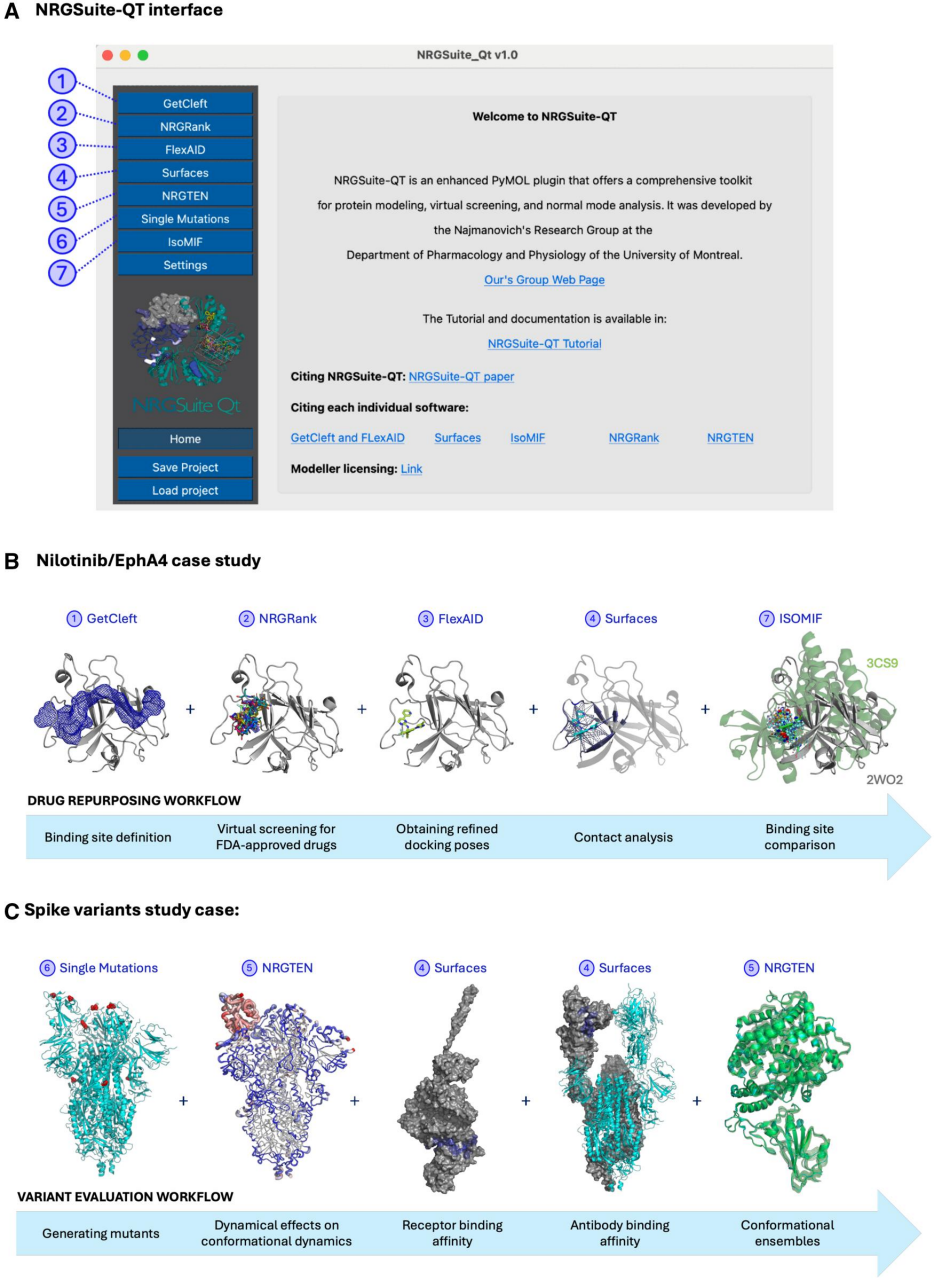
Common uses of NRGSuite. (A) NRGSuite-Qt interface as a PyMOL Plugin. (B) General procedure of the Nilotinib study and the workflow for drug repurposing using the FDA-approved drugs dataset in NRGSuite. (C) Workflow illustrating the study of SARS-CoV-2 Spike variants and their diverse functional effects.

We selected two case studies (see Fig. 1) demonstrating possible workflows that combine multiple tools to reproduce data previously published by Gu *et al.* (2018) and results from two studies published by Teruel *et al.* (2021, 2023). These examples were chosen to demonstrate some of the capabilities of the NRGSuite-Qt to tackle complex problems, such as drug repurposing, predictions of the effects of mutations on protein dynamics, and protein–protein interactions, resulting in outcomes comparable to those of the original publications, but streamlining all analyses sequentially in a single interface. A detailed description of the case studies, including the results obtained, can be found at https://nrg-qt.readthedocs.io/. Here, the focus is on presenting the general workflow for each case study.

### 3.1 Drug repurposing: EphA4

Gu *et al.* (2018) screened a dataset of FDA-approved drugs for inhibitors of the receptor tyrosine kinase Erythropoietin-producing Hepatocellular A4 (EphA4), a molecular target in Alzheimer’s disease. They selected and tested 22 molecules, identifying 5 potential EphA4 inhibitors. Notably, Nilotinib, a known kinase inhibitor, was found to inhibit EphA4-ephrin-A binding at a micromolar scale in a dose-dependent manner. In the NRGSuite-Qt tutorial, we used an EphA4 structure (PDB code: 2WO2), the same one used by *Gu S. et al.* in their study. First, we applied ① GetCleft to identify all cavities in the structure and selected the largest one by volume. We then ran ② NRGRank using its built-in dataset of FDA-approved drugs. Nilotinib appeared among the top 0.1% results, ranked 13th, with a docking score of $-175.54\times 10^{4}$. In a standard virtual screening campaign (Gimeno *et al.* 2019), hundreds of the highest-ranked ligands are typically tested, making Nilotinib a strong candidate for experimental validation in most virtual screening contexts.

Next, we used ③ FlexAID to obtain an accurate docking pose for the Nilotinib/EphA4 complex, followed by ④ Surfaces on the top result to identify key interactions between Nilotinib and EphA4. All interactions cited by *Gu S. et al.*, including Q70, T104, I59, F154, V157, M164, L166, A193, and V159, were also observed in our docking results for Nilotinib, showing high similarity in docking poses. Those findings reinforce that FlexAID captured well the binding mode of Nilotinib, even though the cavity utilized for screening and docking is very big and extends over the whole protein as represented in the first step of the workflow shown in Fig. 1B.

It would be possible to extend this workflow using ⑦ IsoMIF to calculate the similarity between the chemical environments surrounding Nilotinib in the EphA4 binding site and those of Nilotinib bound in other kinases.

In terms of time requirements, we present here some data based on real time required for calculations to give users some idea of what should be expected using a modern standard laptop. The cavity definition step GetCleft was completed in 1 s for the binding site of $1840{Å}^{3}$. Virtual screening of the FDA dataset containing 2619 molecules with NRGRank required approximately 158 s. Refining the binding pose of Nilotinib with FlexAID (1000 chromosomes and 1000 generations, totaling $10^{6}$ energy evaluations) was the most time-consuming step, taking about 193 s. Key interactions between Nilotinib and EphA4 were identified in about 1 s using Surfaces. Finally, IsoMIF, used for binding site comparison, took 52 s when comparing the binding site of EPH4 and Human ABL Kinase bound to Nilotinib (PDB code 3CS9, defining a cavity of $2931{Å}^{3}$). Overall, the total runtime was just under 7 min (405 s). All speed tests were performed on a 10 core M1 Pro chip, and a detailed description of the timing for each step is shown in Table 3, available as supplementary data at *Bioinformatics Advances* online.

### 3.2 Variant evaluation: Spike protein

The SARS-CoV-2 pandemic represented a pivotal moment for global scientific collaboration, driving unprecedented efforts to study the virus and its variants. Among the viral components, the Spike glycoprotein emerged as a key focus due to its high propensity for mutation, with these mutations exerting diverse functional effects. NRGSuite allows us to reproduce results from Teruel *et al.* (2021, 2023, 2025) to evaluate the effects of mutations on Spike’s conformational dynamics, interaction with the receptor angiotensin-converting enzyme 2 (ACE2), as well as immune recognition. Specifically, after employing the ⑥ Single Mutations implementation of Modeler (Eswar *et al.* 2006), ⑤ NRGTEN was applied to evaluate the effects of the D614G mutation, which became predominant in Europe in the summer of 2020, revealing increased flexibility in the closed conformation (PDB code: 6VXX) and rigidity in the open conformation (PDB code: 6VYB), indicating a possible contribution to the occupancy of the open conformation of Spike, needed for receptor binding and cell fusion. The role of this substitution in increasing the open state occupancy was also seen when employing more complex computational methodologies, such as MD (Mansbach *et al.* 2021), and later experimentally validated (Gobeil *et al.* 2021), demonstrating that the ⑤ NRGTEN approach, even though much more simplified, can reach the same conclusions as more sophisticated and computationally costly methods. Given its suitability to high-throughput applications, we can use the same method to explore the dynamical effects of more substitutions. When evaluating mutations N501Y and K417N, introduced in the Alpha and Beta variants, respectively, they exhibited even greater flexibility changes in both states, which could be associated to their selection in these early variants.

Beyond the dynamical consequences of these mutations, there are many protein interactions that are fundamental for receptor binding and immune recognition. ④ Surfaces was employed to analyse per-residue protein-protein interactions between Spike and the receptor ACE2 (PDB code: 6M17), as well as Spike and antibodies, such as the C105 antibody (PDB code: 6XCN). Evaluating the effects of the same two substitutions N501Y and K417N in interactions, we saw that functionally, N501Y enhanced ACE2 binding, in accordance to literature (Starr *et al.* 2020, Laffeber *et al.* 2021, Tian *et al.* 2021, Sergeeva *et al.* 2023), while K417N reduced interactions with C105, potentially diminishing immune recognition, as reported (Greaney *et al.* 2022, Cao *et al.* 2023). Finally, by incorporating protein ensembles generated with ⑤ NRGTEN, our tools are also able to capture conformational variability to provide a dynamic and comprehensive evaluation of these interactions, acknowledging the inherent flexibility of protein structures.

The structural models used for the open and closed states of the Spike protein contain approximately 23 000 atoms (22 507 and 23 739, respectively). Generating a single mutation required 35 s, while evaluating molecular dynamics took an average of 473 s (under 8 min) per structure. For assessing the impact of mutations on ACE2 interactions, generating the N501Y mutation in the B chain of Spike (containing 223 residues) took 2 s, and per-residue interaction evaluation between Spike and ACE2 took 9 s. To analyse the effects of Spike mutations on immune recognition, generating a mutation in a Spike structure with 1259 residues took 17 s. Per-residue interaction evaluation with ④ Surfaces between Spike and the two chains of the C105 antibody (447 residues) required 18 s. Finally, generating the Omicron conformational ensemble took 502 s (under 9 min) to produce 10 conformations of a structure with 195 Spike residues and 596 ACE2 residues. Per-residue interaction evaluation with ④ Surfaces between Spike and ACE2 averaged 5 s per conformer. A table summarizing the execution time for each step is shown in Table 4, available as supplementary data at *Bioinformatics Advances* online.

## 4 Accuracy of the tools

For the task of distinguishing between true binders and decoys, NRGRank was validated using the DUD-E dataset and achieved an average Enrichment Factor of 8 for holo structures (Descoteaux and Najmanovich 2025). Its performance is comparable to the best and most widely used virtual screening software across various datasets. Furthermore, Descoteaux and Najmanovich (2025) shows that NRGRank identifies complementary subsets of true binders from those found with the Glide docking software (Friesner *et al.* 2004) and re-scoring with Glide further enriches top scoring true binders without losing the binders identified by NRGRank. FlexAID was extensively validated for the task of binding mode prediction. The benchmarking studies assessed its ability to predict native ligand poses using the Astex diverse set, with success rate of 66.7% of the native pose being plotted among the TOP poses, with a performance comparable to other widely used docking software (Gaudreault and Najmanovich 2015) as well as prospectively in several instances, most recently with the repurposing of PDB ligands to prevent Ebola replication (Rodríguez-Salazar *et al.* 2024). For the protein–ligand energy decomposition task, Surfaces was validated in the work of Rodríguez-Salazar *et al.* (2024), where it was used to identify the residues with the most favorable interactions. These residues were then mutated, successfully disrupting the interaction between two ligands and Ebola VP35 *in vitro*. Surfaces was also validated for predicting the effect of mutations using the antibody binding data (AB-Bind) dataset, which comprises 1101 mutations in 32 different protein complexes with experimentally determined ΔΔG changes. Its performance was superior or comparable to other commonly used software for structural analysis and energy evaluation, such as bASA, dDFIRE, DFIRE, FoldX, and Rosetta. Additionally, it was validated for the prediction of ACE2 binding for a dataset of 23 SARS-CoV-2 Spike Receptor Binding Domain mutants, demonstrating comparable performance to Free Energy Perturbation (FEP+) while surpassing state-of-the-art techniques such as MM/PBSA methods (Teruel *et al.* 2023). For the dynamical analysis, NRGTEN was extensively benchmarked in our Supplementary Materials, available as supplementary data at *Bioinformatics Advances* online. IsoMIF was validated across multiple datasets, including the Kahraman, SOIPPA, and PDBbind refined datasets, outperforming or matching existing binding site similarity methods. (Chartier *et al.* 2016).

## 5 Conclusions

NRGSuite-Qt represents a significant step toward making state-of-the-art, complex structural computational biophysics workflows accessible to all. This accessibility comes from two key factors: the speed of the methods due to their simplicity or coarse-graining and the intuitive, user-friendly interface. Another pivotal feature of NRGSuite-Qt is the seamless integration of multiple tools within a single platform. This integration allows users to address complex scientific questions, as demonstrated in the case studies presented. By combining capabilities such as virtual screening, docking, the analysis of molecular interactions, and normal mode analysis, NRGSuite-Qt provides a cohesive environment for comprehensive structural studies. These features ensure that NRGSuite-Qt is not only a powerful toolkit but also a catalyst for advancing research in protein engineering and drug discovery.

## Supplementary Material

vbaf129_Supplementary_Data

## Data Availability

NRGSuite-Qt is available under the GNU General Public License (GPL) for macOS, Linux, and Windows. An installation guide and a tutorial featuring different case studies including the two shown here is available at: https://nrg-qt.readthedocs.io/.
