# Supplementary material for: NRGSuite-Qt: a PyMOL plugin for high-throughput virtual screening, molecular docking, normal-mode analysis, the study of molecular interactions, and the detection of binding-site similarities
Source: Bioinform Adv. 2025 Jun 16;5(1):vbaf129. doi: 10.1093/bioadv/vbaf129 (PMC12177131; doi:10.1093/bioadv/vbaf129)
Supplement: vbaf129_Supplementary_Data [file vbaf129_supplementary_data.pdf]

Supplementary Material of "NRGSuite-Qt: A PyMOL plugin for high-throughput virtual screening, molecular docking, normal-mode analysis, the study of molecular interactions and the detection of binding-site similarities"

Gabriel Tiago Galdino[1,†], Thomas DesCôteaux [1,†], Natalia Teruel [1] and Rafael Najmanovich [1,*]

[1]Department of Pharmacology and Physiology, Université de Montréal, Montreal, Canada

[†]These authors contributed equally.

[*]To whom correspondence should be addressed.

May 27, 2025

1 BENCHMARKING ENCOM/NRGTEN WITH THE FLEXAID INTERACTION MATRIX

The possibility of substituting the original 8-atom type matrix employed in ENCoM, based on the LIGIN classes [13], was explored by *Galdino et al.* [5] and validated in the study of dynamics-based ligand efficacy prediction in the μ-opioid receptor. This modified version was implemented in NRGSuite-Qt, and its performance is here evaluated using benchmarks proposed in the original ENCoM paper [4], as well as RNA benchmarks from the work of *Mailhot et al.* [9].

The benchmarks from the original ENCoM paper include the correlation of dynamical signatures with experimentally measured b-factors, conformational change prediction calculated by the cumulative overlap of normal modes, conformational ensembles of NMR structures, and prediction of the effect of mutations on the $\Delta\Delta G$ of folding in protein maturation. *Mailhot et al.* [9] extended these benchmarks to RNA, resulting in a total of 7 benchmarks. All datasets and metrics used in these analyses were constructed and described by *Mailhot* [8] and briefly described in the next subsections.

1.1 *Results*

For the protein b-factors correlation benchmark, we used a dataset of 80 high-resolution protein structures with a maximum length of 300 amino acids, selected from a non-redundant dataset curated by *Kundu et al.* [7], as utilized in the original ENCoM publication. For the RNA b-factors correlation benchmark, we employed a dataset of 38 RNA structures with a resolution of 2.5 Å or better, each up to 300 residues in length and with no missing atoms (benchmark available at [9]).

For each protein or RNA structure, two types of dynamical signatures were calculated, mean-square fluctuations (MSF) and entropic signatures (ES) (equations 1 and 2). ES are dependent

Metric	LIGIN Atypes	SYBYL-based Atypes
Protein b-factors correlation	0.569	0.575
Protein Mutations RSME	1.549	1.527
RNA b-factors correlation	0.446	0.456
Protein overlaps	0.791	0.793
RNA NCO	0.775	0.773
RNA-protein NCO	0.752	0.760
RNA overlaps	0.734	0.728

Table 1: Comparison of metrics for LIGIN atom types, employed by ENCoM, and the SYBYL-based atom types, used for the FlexAID scoring function.

on a thermodynamic scaling factor The ES were calculated for 41 scaling factors ranging from e^{-5} to e^5, and the correlation between the average b-factors of all atoms represented by a bead and their respective fluctuation values in the dynamic signature was calculated. The highest Pearson correlation value (among MSF and all 41 ES) was chosen as representative of the benchmark. We obtained Pearson correlation values of 0.569 for LIGIN atom types and 0.575 for SYBYL-based atom types.

For the protein overlap benchmark, 37 pairs of the same structure representing two forms (apo and holo) with no missing residues and the same length were used from the Protein Structural Change upon Ligand Binding Database (PSCDB) [1] as previously done [4]. Additionally, the RNA overlap benchmark consists of 227 pairs of structures of the same sequence representing two different conformations with a minimum RMSD of 2Å present in the PDB [10]. The cumulative overlap in 5% normal modes from one state to another was calculated in both directions for all pairs of RNA and proteins, and the mean cumulative overlap (equation 6) for both ENCoM versions is reported. The higher the overlap, the more precisely the normal modes calculated using one state, describe the movements required to reach the second state. We obtained an overlap of 0.791 ± 0.15 and 0.793 ± 0.14 for LIGIN and SYBYL-based atom types, respectively, not showing significative difference between both methods.

NMR solution experiments also provide a reliable source of dynamic information. To evaluate the capacity of ENCoM in capturing the movement reported by these experiments, *Mailhot et al.* [9] used Normalized Cumulative Overlap (NCO, equation 7) between the motions apparent from solution NMR ensembles represented by non-rotational-translational correction to principal component analysis (nrt-PCA) and the 5% lowest frequency ENCoM normal modes of RNA alone or Protein/RNA complexes. These benchmarks designed by *Mailhot et al.* [9] contain 313 solution NMR resolved structural ensembles of RNA with at least two different states and 20 solution NMR ensembles of RNA/protein complexes with a maximum of 70% sequence variation, respectively. As with the protein overlap benchmark, a higher value is better. For the RNA-protein complexes test, we obtained 0.760 correlation for SYBYL-based atom types and 0.752 for LIGIN atom types. For RNA molecules alone, we obtained 0.773 for SYBYL-based atom types and 0.775 for LIGIN atom types. This is the only case where LIGIN atom types still show an advantage, although very small, perhaps reflecting the fact that the SYBYL-based atom type parameters were originally developed to study ligand-protein complexes and RNA interactions were not considered [6]. The optimization of pairwise interaction pseudo-energies in future studies may further improve performance, particularly for the analysis of complexes involving RNA and protein-protein interactions.

The original ENCoM [4] paper demonstrated that the predicted vibrational entropy of wild-type and mutants (ΔS_{vib}, equation 3) can be used as a linear approximation of the experimentally measured $\Delta\Delta G$ of folding induced by mutations. To evaluate the performance of ENCoM in predicting the effect of mutations on the stability of proteins, the authors performed a linear fitting between ΔS_{vib} and the experimentally measured $\Delta\Delta G$ of mutants from the ProTherm database [12] and reported the root mean square error (RMSE). This benchmark dataset contains 299 models of the mutations reported in the ProTherm database, generated from their structures available in the PDB using Modeller [3]. The smaller the reported RMSE, the better the performance of the method. The new vibrational entropy implemented in NRGTEN depends on a thermodynamic scaling factor. For each scaling factor listed in the b-factors benchmark (41 scaling factors ranging from e^{-5} to e^{5} as described above), we calculated the ΔS_{vib} for all mutants and then computed the RMSE against their experimentally measured $\Delta\Delta G$. We selected the scaling factor that produced the lowest RMSE as representative of this benchmark. We obtained an RMSE of 1.549 (scaling factor $e^{-0.5}$) for the LIGIN atom types model and 1.527 (scaling factor e^{-5}) for SYBYL-based atom types. Given the differences in the number and range of parameters for each of the two atom type schemes, it is natural to expect different thermodynamics scaling factors.

Table 1 summarizes all the results. The analysis of the benchmarks shows that utilizing the SYBYL-based atom types with ENCoM offers improvements in predicting protein dynamics, particularly for mutation stability prediction. Notably, the improvement in RNA b-factors correlation suggests that the new atom-type matrix is more effective in capturing RNA dynamics than the original matrix. The adoption of SYBYL-based atom types in NRGTEN generally enhances the accuracy of dynamical predictions across both protein and RNA structures, with no significant losses in performance. Since it has also been validated for the prediction of ligand efficacy in GPCRs by *Galdino et al.* [5], this version was implemented as the default version of ENCoM in the NRGSuite-Qt. With the adoption of the SYBYL-based atom types, FlexAID, NRGRank, ENCoM and Surfaces, all utilize the same atom-type scheme and pairwise atom type pseudo-energy scoring matrix.

1.2 *Metrics*

1.2.1 *Dynamical signatures and Vibrational Entropy*

For the *Protein b-factors* correlation and the *RNA b-factors correlation* benchmarks the metrics used are:

Mean-Square-Fluctuations (MSF): The Mean Square Fluctuations vector shows the overall flexibility of each bead i in the Elastic Model by the sum of the contributions of each the non-trivial normal normal mode E_n in all directions. The 6 first normal mode represents the rotational and translational movements of the system while the 3N-7 left represents vibrational movements, where N is the total of nodes in the system.

$$\text{MSF}_i = \sum_{n=7}^{3N} E_{n,i,x}^2 + E_{n,i,y}^2 + E_{n,i,z}^2 \tag{1}$$

Entropic Signatures (ES): The Entropic signatures derivate from by introducing a temperature depended factor $S_{\text{vib},n}$ representing the vibrational entropy of the system entropy

$$\text{ES}_i = \sum_{n=7}^{3N} S_{\text{vib},n} \left(E_{n,i,x}^2 + E_{n,i,y}^2 + E_{n,i,z}^2 \right) \tag{2}$$

where ES_i is the entropic mean square fluctuation for the ith bead in the elastic network, N is the total number of beads, $E_{n,i}$ is the movement of the bead in the nth normal mode, and $S_{\text{vib},n}$ is the vibrational entropy associated with the nth normal mode.

$$S_{\text{vib},n} = \frac{\beta \nu_n}{e^{\beta \nu_n} - 1} - \ln(1 - e^{-\beta \nu_n}) \tag{3}$$

$$\nu_n = \frac{1}{2\pi}\sqrt{\lambda_n} \tag{4}$$

where λ_n is the vibrational frequency associated to the eigenvalue of the nth normal mode and β a thermodynamic scaling factor.

For the *Protein RSME* is calculated by getting the total $S_{\text{vib},n}$ in equation (3) over all beads of the wt and calculate the difference to the mutant. This value is than plotted against the experimental measure

1.2.2 *Cumulative Overlap*

The overlap metric quantifies the similarity between an normal mode $\vec{E}_n$, predicted from the starting conformation, and the displacement vector obtained from the difference in coordinates between the target and input conformations $\vec{R}$, following their superposition.

$$\text{overlap}(\vec{E}_n, \vec{R}) = \frac{\left|\vec{E}_n \cdot \vec{R}\right|}{\|\vec{E}_n\|\|\vec{R}\|} \tag{5}$$

The cumulative overlap for the N-slowest normal modes can be obtained by:

$$\text{CO} = \sqrt{\sum_{n}^{N} \text{overlap}(\vec{E}_n, \vec{R})^2} \tag{6}$$

1.2.3 *Normalized Cumulative Overlap*

For the Normalized Cumulative Overlap matric we applied the non-rotational-translational Principal Component Analysis (nrt-PCA) introduced by *Mailhot et al.* [9]. It is an extension of standard PCA designed to remove rotational and translational degrees of freedom from the principal components of molecular conformational ensembles. While traditional PCA is useful for analyzing dominant motions in a system, it can inadvertently capture global rotations and translations, which may obscure the intrinsic internal motions of the structure. nrt-PCA addresses this by first computing standard PCs after superimposing all conformations to a reference model. Then, using Gram-Schmidt orthonormalization [2], the PCs are adjusted by removing contributions from the six rotational-translational modes of the reference structure. This ensures that only meaningful internal motions are retained while maintaining a Cartesian representation, making the method particularly useful for biomolecular dynamics studies. The corrected PCs are reordered based on their adjusted variance contribution, allowing for a more accurate comparison of structural dynamics without interference from trivial motions.

The Normalized Cumulative Overleap between the first N normal modes and the first M principal components (PCs) can be calculated using:

$$\text{NCO} = \sum_{j=1}^{M} \left[v_j \sqrt{\sum_{i=1}^{N} \text{overlap}(\vec{E}_i, \vec{PC}_j)^2} \right] \tag{7}$$

where:

- $\vec{E}_i$ represents the **normal mode** i.
- $\vec{PC}_j$ is the **principal component** j.
- v_j denotes the **proportion of variance** explained by component j.

The NCO ensures a value between 0 and 1, as both the normal modes and the principal components are orthogonal with respect to themselves, and v_j sums to 1 over all the components.

2 DISTRIBUTION OF TANIMOTO SCORE OF BINDING-SITE SIMILARITIES

To determine whether two binding sites are significantly similar or different, we selected the dataset of all 102 targets from the DUD-E database [11]. For each ligand/target complex, we defined the cavity around the ligand using GetCleft to represent the binding site. This cavity is saved and the complex is then protonated using PyMOL's "add hydrogens" functionality. We then selected all combinations of pairs of complexes and ran IsoMIF on each pair of cavities using the ligands as references. We calculate the distribution of Tanimoto scores of binding-site similarities for pairs of targets from different families as classified by *Mysinger et al.* (2012) [11]. We also calculate the distribution for pairs of targets from different families. Target families in DUD-E include Other Enzymes (36), Kinases (26), Proteases (15), Nuclear Receptors (11), GPCRs (5), Miscellaneous (5), Cytochrome P450 (2) and Ion Channels (2). Figure 1 shows the distribution of Tanimoto scores of binding-site similarity for all 102 target, those in different or the same family, and only for kinases as the largest family with evolutionarily related proteins.

The mean Tanimoto coefficient for all pairs or pairs from different families is 0.21, and both distributions have a similar shape. This is not surprising as overall, most proteins in the dataset are not evolutionarily related even if belonging to the same family. For proteins of the same family, the mean is shifted to 0.23, indicating a higher similarity between proteins of the same family. The distribution is significantly shifted, with a mean value of 0.27, when considering only the kinase family, which can be explained by the fact that this family is known to have high similarity and promiscuity of ligands among different members of the family. When the user of the NRGSuite-Qt performs the detection of binding-site similarities, the Tanimoto score of binding-site similarity is plotted in relation to the distribution of Tanimoto scores across different families and serves as the basis to calculate a p-value and Z-score for the observed similarities.

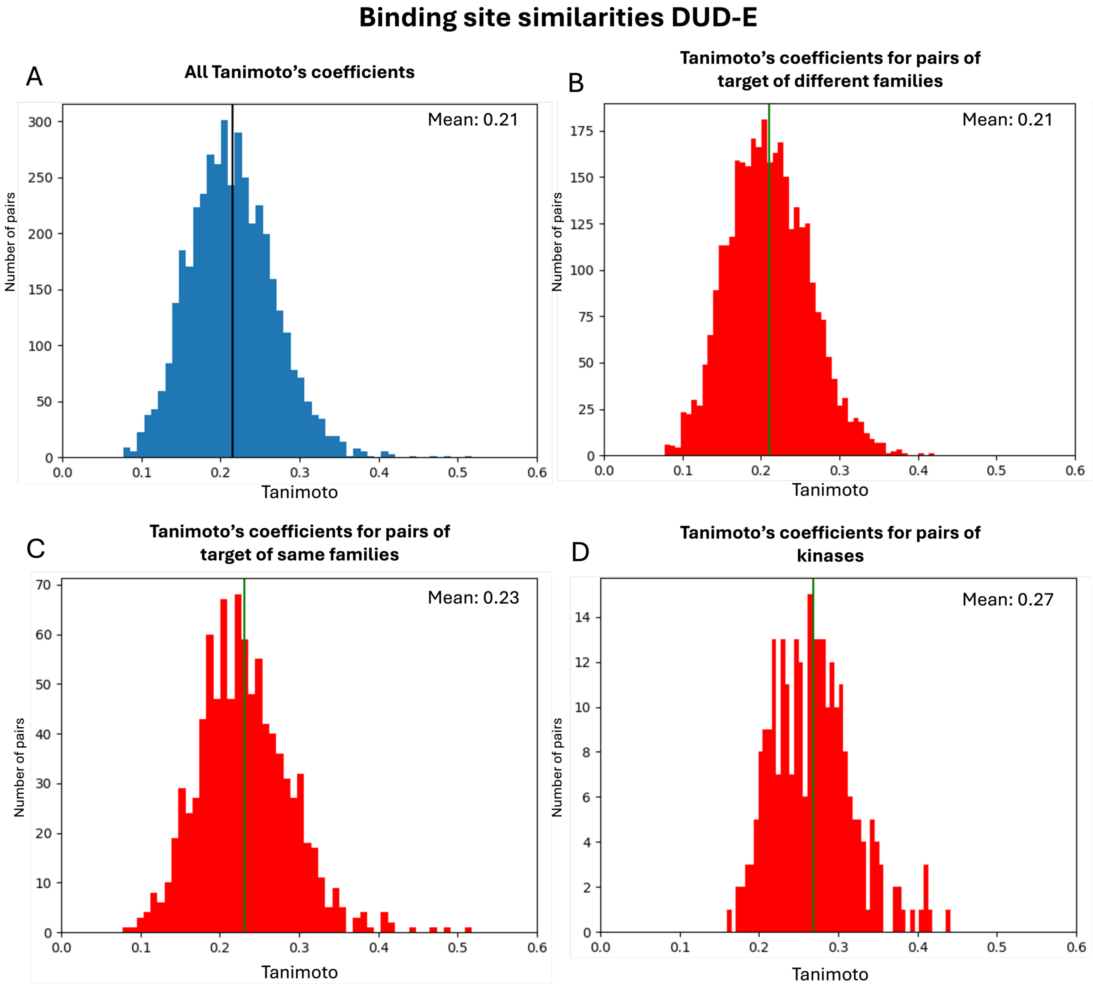

Figure 1: Tanimoto's coefficient distribution for all DUD-E targets. (A) Tanimoto distribution for all pairs of targets. (B) Tanimoto distribution for target pairs of different families. (C) Tanimoto distribution for target pairs of the same families. (D) Tanimoto distribution for kinase pairs.

3 COMPARISON BETWEEN NRGSUITE AND NRGSUITE-QT

**Feature**	**NRGSuite**	**NRGSuite-Qt**
**User Interface**	Tkinter	Qt
**Docking Software**	FlexAID	FlexAID: implementation focused in obtaining docking poses
**Cavity Detection**	GetCleft	GetCleft: same functionality
**Virtual Screening**	Not available	NRGRank: ultra-massive screening
**Interaction Analysis**	Not available	Surfaces: protein-ligand and protein-protein interactions
**Normal Mode Analysis**	Not available	NRGTEN: dynamical signatures and entropic analysis
**Binding-Site Similarity**	Not available	IsoMIF: binding-site molecular interaction field analysis
**Mutational Studies**	Not available	Modeller-based single mutant generation
**Supported Platforms**	Windows, Linux, MacOS	Windows and MacOS

Table 2: Comparison of NRGSuite and NRGSuite-Qt.

4 TIMING OF THE CASE STUDIES

Table 3: EphA4 Drug Repurposing Execution times

**Task**	**Software**	**Execution Time (s)**
Generate binding site (EphA4)	① **GetCleft**	0.3
Screen FDA Molecules	② **NRGRank**	158
Obtain docking pose for Nilotinib	③ **FlexAID**	193
Identify key interactions between EphA4 and Nilotinib	④ **Surfaces**	1
Generate binding site (3GP0 and 3CS9)	① **GetCleft**	1
Binding site similarity of EphA4 and 3GP0/3CS9	⑦ **IsoMIF**	52

Table 4: Protein-Protein analysis (Spike)

Dynamical effects of mutations		
**Task**	**Software**	**Execution Time (s)**
Generate mutations (D614G/K417N/N501Y)	6 Single mutations	35
Evaluate the effect of the mutation on dynamics	5 NRGTEN	473
**Effects of mutations on ACE2 interactions**		
**Task**	**Software**	**Execution Time (s)**
Generate mutation (N501Y)	6 Single mutations	2
Per-residue interaction evaluation (Spike RBD with ACE2)	4 Surfaces	9
**Effects of mutations on immune recognition**		
**Task**	**Software**	**Execution Time (s)**
Generate mutation (K417N)	6 Single mutations	17
Per-residue interaction evaluation (Spike and antibody C105)	4 Surfaces	18
**Effect of structural variability in Omicron on Spike/ACE2 binding**		
**Task**	**Software**	**Execution Time (s)**
Generate conformational ensemble (simulate structural variability)	5 NRGTEN (Conformational ensembles)	502
Per-residue interaction evaluation (Spike RBD with ACE2)	4 Surfaces	5

REFERENCES

[1] Takayuki Amemiya, Ryotaro Koike, Akinori Kidera, and Motonori Ota. Pscdb: A database for protein structural change upon ligand binding. *Nucleic Acids Research*, 40, 1 2012.

[2] George Arfken. *Mathematical Methods for Physicists*. Academic Press, 1985.

[3] Narayanan Eswar, Ben Webb, Marc A Marti-Renom, MS Madhusudhan, David Eramian, Min yi Shen, Ursula Pieper, and Andrej Sali. Comparative protein structure modeling using modeller modeling structure from sequence 5.6.1, 2006.

[4] Vincent Frappier and Rafael J. Najmanovich. A coarse-grained elastic network atom contact model and its use in the simulation of protein dynamics and the prediction of the effect of mutations. *PLoS Computational Biology*, 10, 2014.

[5] Gabriel T. Galdino, Olivier Mailhot, and Rafael Najmanovich. Understanding and predicting ligand efficacy in the mu-opioid receptor through quantitative dynamical analysis of complex structures. *Journal of Chemical Information and Modeling*, 64:8549–8561, 11 2024.

[6] F Gaudreault and RJ Najmanovich. Flexaid: Revisiting docking on non-native-complex structures. *Journal of Chemical Information and Modeling*, 55(7):1323–1336, 2015.

[7] Sibsankar Kundu, Julia S. Melton, Dan C. Sorensen, and George N. Phillips. Dynamics of proteins in crystals: Comparison of experiment with simple models. *Biophysical Journal*, 83:723–732, 2002.

[8] Olivier Mailhot. *Predicting biomolecular function from 3D dynamics: sequence-sensitive coarse-grained elastic network model coupled to machine learning*. PhD thesis, Université de Montréal, Montréal, August 2022. Supervisors: Rafael Najmanovich, François Major.

[9] Olivier Mailhot, Vincent Frappier, François Major, and Rafael J. Najmanovich. Sequence-sensitive elastic network captures dynamical features necessary for mir-125a maturation. *PLoS Computational Biology*, 18, 12 2022.

[10] Olivier Mailhot and Rafael Najmanovich. The nrgten python package: an extensible toolkit for coarse-grained normal mode analysis of proteins, nucleic acids, small molecules and their complexes. *Bioinformatics*, 37:3369–3371, 10 2021.

[11] Michael M. Mysinger, Michael Carchia, John J. Irwin, and Brian K. Shoichet. Directory of useful decoys, enhanced (dud-e): Better ligands and decoys for better benchmarking. *Journal of Medicinal Chemistry*, 55:6582–6594, 7 2012.

[12] Rahul Nikam, A. Kulandaisamy, K. Harini, Divya Sharma, and M. Michael Gromiha. Prothermdb: Thermodynamic database for proteins and mutants revisited after 15 years. *Nucleic Acids Research*, 49:D420–D424, 1 2021.

[13] Vladimir Sobolev, Rebecca C Wade, Gert Vriend, and Marvin Edelman. Molecular docking using surface complementarity, 1996.
